# Assessment of awareness, practices, perceptions, and satisfaction of telepsychiatry among mental healthcare providers in Saudi Arabia

**DOI:** 10.3389/fpsyt.2025.1426998

**Published:** 2025-05-02

**Authors:** Omar A. Alshaikhi, Lujain A. Aldarsi, Ibrahim Abdullah A. Basfar, Alwaleed Alshehri, Raafat M. Shuqdar, Ramy Mohamed Ghazy, Mahmoud Essam Elrggal

**Affiliations:** ^1^ King Abdulaziz Hospital & Oncology Center, Ministry of Health, Jeddah, Saudi Arabia; ^2^ Alamal Mental Health Hospital, Ministry of Health, Medinah, Saudi Arabia; ^3^ Eradah and Mental Health Complex, Jeddah, Saudi Arabia; ^4^ Abha Mental Health Hospital, Ministry of Health, Abha, Saudi Arabia; ^5^ College of Medicine, Taibah University, Madina, Saudi Arabia; ^6^ Tropical Health Department, High Institute of Public Health, Alexandria University, Alexandria, Egypt; ^7^ Family and Community Medicine Department, College of Medicine, King Khalid University, Abha, Saudi Arabia; ^8^ Department of Pharmacology and Toxicology, Umm al-Qura University, Mecca, Saudi Arabia

**Keywords:** telemedicine, telepsychiatry, tele-mental health, psychiatric service, mental healthcare providers

## Abstract

**Background:**

Telepsychiatry, a global method for mental health services, has gained global attention, especially in the corona-virus diseases 2019 (COVID-19) era. It uses electronic communication and information technologies for remote psychiatric care, with synchronous modalities involving real-time interactions and asynchronous modalities allowing indirect communication. This study aimed to assess the awareness, practices, perceptions, and satisfaction of mental healthcare providers (MHPs) in the Kingdom of Saudi Arabia (KSA) regarding telepsychiatry utilization.

**Method:**

This online, survey-based cross-sectional study included MHPs, both physicians and non-physicians, working in public and private mental health services across various regions of KSA. The study questionnaire was distributed using Google Forms via email and other popular social media platforms (including WhatsApp, Twitter, Telegram, and Facebook). The questionnaire was developed to assess the personal and socio-demographic characteristics of the participants, as well as their awareness, practices, perceptions, and satisfaction regarding telepsychiatry. Participants were recruited using convenience and snowball sampling techniques.

**Results:**

Out of the 500 MHPs enrolled in the study, 52.2% were under 30 years, 52.6% were male, and 54.8% were single. Participants were from five regions: Central (27.6%), Western (22.6%), Eastern (22.0%), Southern (16.8%), and Northern (11.0%). Professionally, 33.8% were psychiatric residents, 21.8% were psychologists, 19.2% were social workers, and 12.6% each were psychiatric consultants and specialists. Of the study participants, 73.8% demonstrated awareness of telepsychiatry. More than three-fifths (63.7%) had previous practical experience. Among those with experience, 82.9% reported telepsychiatry practice durations of 3 years or less. Perception and satisfaction percentage scores for different domains indicated high perception regarding the advantages and disadvantages (62.6% ± 13.9) and improved patient access (75.2% ± 17.4). However, lower satisfaction scores were observed for MHPs’ access satisfaction (46.6% ± 11.7) and practice satisfaction (57.6% ± 9.6).

**Conclusion and recommendation:**

MHPs in KSA exhibit high awareness but engage in telepsychiatry practice to a lesser extent. They have a good perception and are satisfied with their telepsychiatry practice. The study recommends that policymakers and stakeholders in KSA should prioritize building the capacities of MHPs in telehealth. Expanding and scaling up awareness activities are essential to improve digital literacy and telehealth practices among MHPs

## Inroduction

According to the Global Burden of Disease Study 2019 (GBD 2019), it has been estimated that mental health diseases affect approximately 555.44 million patients, ranking as the eighth contributor to disability burden in Asia in 2019 (5). The Saudi National Mental Health Survey (SNMHS) reported that the twelve-month prevalence of mental disorders is estimated to be 20.2% in the Kingdom of Saudi Arabia (KSA), which is considered relatively high compared to other high-income countries (6). It has been estimated that two out of five Saudi young adults meet the diagnostic criteria for a mental health disorder during their lifetime (7). Specifically, a high prevalence of stress, depression, and anxiety has been reported among university students (8), yet only 5% of those affected seek mental health consultation (7).

Despite the substantial burden of mental health diseases, many considerable barriers were demonstrated to prevent patients from seeking mental health consultation (9). These include the lack of knowledge and negative attitude about mental health consultations among patients, lack of perceived need, mental stigma and confidentiality concerns (7, 10, 11). Additional barriers encompassed cultural and religious beliefs, financial barriers, transportation difficulties and the limited availability and access to psychiatric health services (11, 12). Eventually, delayed management of these health problems is associated with detrimental effects not only on the patient’s quality of life and educational achievement but also on the overall societal health and economic status (13). To enhance access to mental healthcare and reduce the treatment gap, these obstacles should be recognized and addressed (14).

Technological advances in healthcare have expanded the range of options available for diagnosing, treating, and monitoring patients who seek remote care (15). The integration of information systems and technological advances led to the renaissance of healthcare delivery (16). Telemedicine generally refers to the use of technology and telecommunication to assist in medical practice. It has a wide range of uses, including online patient consultations, remote control, telehealth nursing, and remote physical and psychiatric rehabilitation (17). According to the World Health Organization, telemedicine is defined as a means of improving the health of individuals and communities, preventing diseases and accidents, and providing healthcare services by using remote, valid information communication methods. This, together with the continuous training of healthcare staff on the use of information and communication technologies (ICT) (18). On the other hand, some challenges and limitations exist hindering the widespread use of telehealth services, such as reimbursement issues, lack of direct eye contact with patients, the necessity of security software development for patient information protection and stable access to video conferencing (19).

The increased demand for mental healthcare services during and after the coronavirus disease 2019 (COVID-19) pandemic paved the way for significantly increased adoption of digital health and telepsychiatry (20, 21). Telepsychiatry health is demonstrated as the remote sharing of patient information via electronic communication systems such as video conferencing, landlines and mobile phone lines, computer-based internet tools, home-based telehealth phones and additional devices, focusing on the psychiatric aspects to improve the mental health of patients (22, 23). Telepsychiatry refers to the use of electronic communication and information technologies to deliver or support psychiatric care remotely. It can be categorized into synchronous and asynchronous modalities. Synchronous telepsychiatry involves real-time interactions between the patient and clinician, such as video consultations. In contrast, asynchronous telepsychiatry allows for indirect or time-delayed communication, where information is shared and reviewed at different times (1). This adoption is a safe and accepted option for both mental healthcare providers (MHPs) and patients, where MHPs can safely deliver their services through digital platforms and allow patients to receive the care needed while at home (4, 24, 25). The telepsychiatry approach overall improved the quality and availability of care (19).

The expansion of telepsychiatry reflects the progress that has been made in the health field, improving access and quality of care (19). It offers several benefits for both patients and providers. It saves time and effort for clinicians and healthcare providers. Moreover, it improves patients’ access to care, especially for those living in remote areas, alleviates the feeling of stigma for patients seeking mental healthcare, and improves patients’ compliance with treatment and continuity of care (19, 20, 24). Additionally, it allows better healthcare-related choices, increases the quality and performance of emergency services, and reduces the time to reach final diagnoses. Finally, it improves the efficiency of healthcare-related services due to cost reduction for both providers and patients by optimizing clinical procedures and minimizing expenses for transport to hospitals and clinics (17, 26).

Various advancements in telemedicine have evolved in KSA. The Ministry of Health (MOH) has adopted a new strategy to maximize the effectiveness of healthcare services and to enhance patient healthcare accessibility regardless of their geographic location (27). Numerous telehealth applications have been introduced, such as Seha, Tawakkalna, Mawid, Tabaud, and Tataman, between 2018 and 2020, to improve the provision of healthcare services in KSA (28). However, cultural and religious factors pose significant challenges to the implementation of telepsychiatry, as many psychiatrists remain skeptical about its outcomes, and clinicians express dissatisfaction with the service, reducing their willingness to adopt telemedicine (2). Additional obstacles include a shortage of qualified experts to implement the technology, deficiencies in essential ICT infrastructure, and the absence of effective strategies and plans for telemedicine implementation. Furthermore, some healthcare providers lack ICT skills, limiting their ability to adopt this innovation (2, 3).

To achieve and establish an effective telepsychiatry service, MHPs must have a good amount of knowledge and positive attitudes toward its use and must be skilled in using ICT (29). The reported benefits of telepsychiatry practice have motivated researchers to conduct studies aiming at improving telepsychiatry practice and understanding perceptions and barriers among MHPs and patients to expand and scale up the service and improve the chance to officially integrate it into public and private mental health services. This study aimed to assess the awareness of telepsychiatry among MHPs in the KSA as well as their perceptions and satisfaction with telepsychiatry service.

## Subjects and methods

### Study design and setting

This descriptive cross-sectional study was conducted to assess the awareness, practices, perceptions, and satisfaction regarding telepsychiatry among MHPs in the KSA.

### Sample size and study population

We assumed that 5% of MHPs are aware of telepsychiatry, requiring a minimum sample of 199 to achieve 80% statistical power to detect a small-to-moderate effect size (g = 0.1) at a 0.05 significance level. To ensure an adequate sample for assessing user satisfaction, we increased this by 2.5. For evaluating satisfaction with telepsychiatry, the required sample size, as determined by G*Power, was 166 participants. This calculation maintains 80% power to detect a small-to-moderate effect size (g = 0.1) at a 0.05 significance level, assuming a constant proportion of 0.66 of healthcare providers accepting tele-mental health services (4). Inclusion and exclusion criteria: The study included licensed MHPs (physicians, psychologists, and social workers) actively delivering psychiatric health services in public and private hospitals and centers across various regions of KSA. KSA is administratively divided into five primary regions, each encompassing multiple provinces. Central Region (Al-Riyadh), Western Region (Al-Madinah, Makkah, and Tabuk), Eastern Region (Al-Sharqiyah), Southern Region (Al-Jazan, Asir, and Najran), Northern Region (Al-Hudud Al-Shamaliyah, Al-Jawf, and Tabuk). Participants were recruited from diverse geographic regions and practice settings to ensure a broad representation of perspectives. Experience, perception, perceived access, and satisfaction were assessed only for those who were practicing telepsychiatry. Incomplete or inadequately filled survey responses were excluded to maintain data quality. Participation was voluntary, and informed consent was obtained from all respondents.

### Data collection instrument

The survey instrument comprised three sections of closed-ended questions:

1. Socio-demographic information: Items included age, gender, marital status, work region, profession, workplace, and years of experience.
2. Awareness and experience of telepsychiatry: This section queried participants on their prior knowledge about telepsychiatry.
3. Practice of telepsychiatry: Platform used, duration of telepsychiatry practice, total number of treated patients, number of patients treated in the past year, number of sessions/months, self-education of telepsychiatry, received professional training in telepsychiatry, participated in research related to telepsychiatry.
4. Perceptions and satisfaction with telepsychiatry: This section contained 30 statements divided into four specific domains:

a. Perceived advantages and disadvantages of telepsychiatry (6 statements).
b. Perceived patient access to telepsychiatry services (3 statements).
c. Perceived MHP’s access to telepsychiatry services (6 statements).
d. Overall satisfaction with telepsychiatry practice (15 statements).

Responses were recorded on a 5-point Likert scale (0 = strongly disagree, 1 = disagree, 2 = neither agree nor disagree, 3 = agree, 4 = strongly agree), with reversed scoring applied to negatively phrased statements. Reversed statements are marked in **Table 1**. For analysis, we collapsed responses as follows: 0 and 1 (disagree) and 3 and 4 (agree). The questionnaire was developed based on a comprehensive review of the existing literature on telepsychiatry and was refined following expert consultations with 2 consultant psychiatrists to ensure clarity, relevance, and content validity. The content and face validity of the tool were assessed by a group of consultant psychiatrists. They also examined the tool for clarity, significance, comprehensiveness, wording, and understanding. Their suggestions and recommendations were taken into consideration.

### Pilot testing

A pilot test was conducted on 30 MHPs (comprising 10 psychiatrists, 10 social workers, and 10 psychologists) to evaluate the clarity of the items and to estimate the time required for completion. Feedback from this pilot test was used to make the final revised survey. Those who participated in the pilot test were excluded from the main study sample.

#### Survey distribution

Data was collected through an online questionnaire that was distributed using Google Forms via e-mails and other popular social media platforms (including WhatsApp, Twitter, Telegram, and Facebook) that are commonly used by MHPs in KSA between August 1^st^, 2023, to August 31^st,^ 2023. The survey was sent again and reposted every week by e-mail or on social media platforms. Only one response was allowed from each participant. These distribution methods were selected for their wide reach and cost-effectiveness, enabling rapid data collection.

#### Ethical considerations

The study was approved by the Research Ethics Committee at Umm Al-Qura University (approval no: UMZX060622). The importance and purpose of the study, as well as the potential risks and benefits of participation, were clearly explained to prospective participants before their consent was obtained. Only consenting MHPs were asked to participate and fill out the questionnaires. The anonymity, confidentiality, and privacy of the participating MHPs were assured; they were informed about the possibility of withdrawing voluntarily at any time without any consequences.

#### Statistical analysis

Statistical analysis was conducted using the Statistical Package for Social Sciences (SPSS) version 24.0. Categorical variables were summarized using frequencies and percentages. Data normality was assessed through histogram visualization and the Kolmogorov-Smirnov test. For quantitative variables, mean and standard deviation (SD) were used to describe normally distributed data, whereas median and interquartile range (IQR) were applied for skewed distributions. The reliability of the study instruments was assessed using Cronbach’s alpha, where a value of ≥ 0.70 was considered acceptable, indicating good internal consistency. For bivariate analysis, Pearson’s chi-squared test was employed to assess associations between qualitative variables. To compare mean differences between two groups, an independent t-test was used for normally distributed data, whereas the Mann-Whitney U test was applied for non-normally distributed data. A p-value < 0.05 was considered statistically significant across all tests.

## Results

### Participants’ characteristics

The questionnaire was distributed to 638 MHPs, of whom 500 fully completed the survey with no missing data (response rate = 78.3%). The study sample included 52.2% of participants under 30 years old, 52.6% were male, and 54.8% were single. Participants were distributed across five regions: Central (27.6%), Western (22.6%), Eastern (22.0%), Southern (16.8%), and Northern (11.0%). Professionally, 33.8% were psychiatric residents, 21.8% were psychologists, 19.2% were social workers, and 12.6% each were psychiatric consultants and specialists. Physicians accounted for 59.0%, while non-physicians made up 41.0%. Most worked in public settings (65.0%), followed by private (15.4%) and both sectors (19.6%). Experience levels ranged from 0–2 years (35.6%) to 11+ years (18.6%), with a median (IQR) of 4.0 (2.0 – 8.75) years **Table 1**.

**Table 1:** Sociodemographic and professional characteristics of study participants (n = 500).

Characteristic (n = 500)	Level	Total sample n (%)
Age	< 30 years	261 (52.2)
	30-39 years	147 (29.4)
	> 40 years	92 (18.4)
Gender	Male	263 (52.6)
	Female	237 (47.4)
Marital Status	Single	274 (54.8)
	Married	226 (45.2)
Work region	Central Region	138 (27.6)
	Western Region	113 (22.6)
	Eastern Region	110 (22.0)
	Southern Region	84 (16.8)
	Northern Region	55 (11.0)
Profession	Psychiatric consultant	63 (12.6)
	Psychiatric specialist	63 (12.6)
	Psychiatric resident	169 (33.8)
	Psychologist	109 (21.8)
	Social worker	96 (19.2)
Physicians	Physician	295 (59.0)
	Non-physician	205 (41.0)
Place of work	Public	325 (65.0)
	Private	77 (15.4)
	Both	98 (19.6)
Years of experience in psychiatry	0-2 years	178 (35.6)
	3-5 years	155 (31.0)
	6-10 years	74 (14.8)
	11+ years	93 (18.6)
	Median (IQR)	4.0 (2.0 – 8.75)

**Figure 1:**
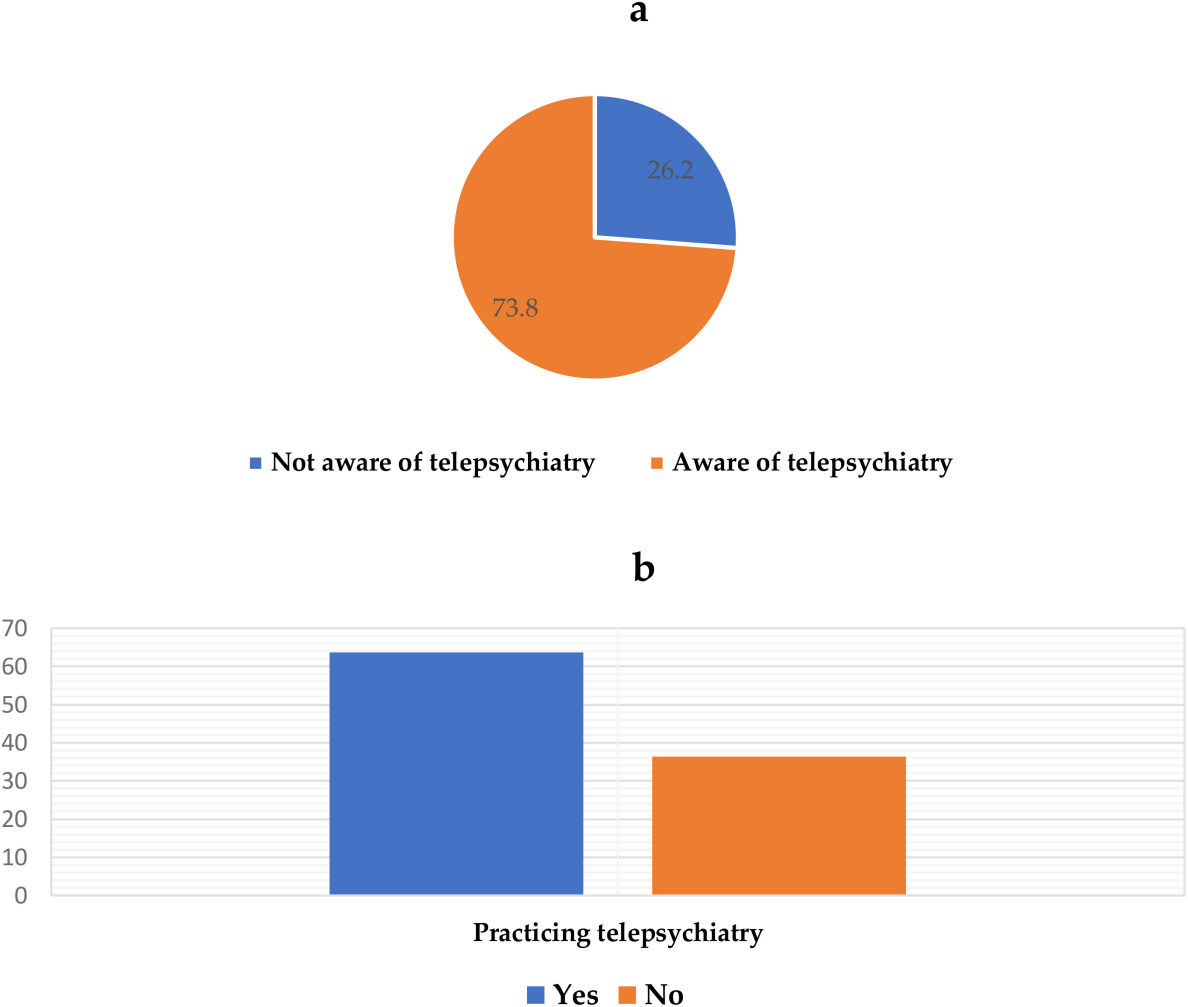
Participants’ prior awareness **(a)** and prior practice of telepsychiatry **(b)**.

Of the 500 surveyed MHPs, 369 (73.8%) were aware of telepsychiatry and 235 (63.7%) of them already practised telepsychiatry **Figure 1**.

Age was not significantly associated with prior awareness of telepsychiatry. However, the highest proportion of MHPs who were unaware of telepsychiatry was among those under 30 years old (78; 29.9%). Gender also showed no significant association, with a slightly higher percentage of females lacking awareness (67; 28.3%) compared to males (64; 24.3%). Marital status revealed a non-significant trend (p = 0.060), with single MHPs (81; 29.6%) more likely to lack awareness than married participants (50; 22.1%). Work region, however, showed a significant association (p < 0.001), with the highest absence of awareness in the Northern Region (22; 40.0%). Although awareness was lower among those working in the private sector (22; 28.6%), neither workplace nor profession showed a significant association. Notably, years of experience were significantly linked to awareness (p = 0.030), with the highest lack of awareness among MHPs with 0–2 years of experience (60; 33.7%). Participants who were unaware of telepsychiatry were excluded from further analysis, leaving 369 MHPs for subsequent assessments **Table 2**.

**Table 2:** Characteristics of MHPs and their association with awareness of telepsychiatry.

Characteristic (n = 500)	Level	Unaware of telepsychiatry n (%) 131 (26.2)	Aware of telepsychiatry n (%) 369 (73.8)	p-value
Age	< 30 years	78 (29.9)	183 (70.1)	*p* = 0.145
	30-39 years	32 (21.8)	115 (78.2)	
	> 40 years	21 (22.8)	71 (77.2)	
Gender	Male	64 (24.3)	199 (75.7)	*p* = 0.318
	Female	67 (28.3)	170 (71.7)	
Marital Status	Single	81 (29.6)	193 (70.4)	*p* = 0.060
	Married	50 (22.1)	176 (77.9)	
Work region	Central region	19 (13.8)	119 (86.2)	*p* < 0.001*
	Western region	38 (33.6)	75 (66.4)	
	Eastern region	37 (33.6)	73 (66.4)	
	Southern region	15 (17.9)	69 (82.1)	
	Northern region	22 (40.0)	33 (60.0)	
Profession	Psychiatric consultant	15 (23.8)	48 (76.2)	*p* = 0.781
	Psychiatric specialist	15 (23.8)	48 (76.2)	
	Psychiatric resident	44 (26.0)	125 (74.0)	
	Psychologist	27 (24.8)	82 (75.2)	
	Social worker	30 (31.3)	66 (68.8)	
Physicians	Physician	74 (25.1)	221 (74.9)	*p* = 0.462
	Non-physician	57 (53.8)	148(72.2)	
Place of work	Public	87 (26.8)	238 (73.2)	*p* = 0.609
	Private	22 (28.6)	55 (71.4)	
	Both	22 (22.4)	76 (77.6)	
Years of experience in psychiatry	0-2 years	60 (33.7)	118 (66.3)	*p* = 0.030 ^*^
	3-5 years	31 (20.0)	124 (80.0)	
	6-10 years	19 (25.7)	55 (74.3)	
	11+ years	21 (22.6)	72 (77.4)	
	Median (IQR)	3.0 (1.5- 8.0)	4.0 (2.0 – 9.0)	*p* = 0.031^c^

Chi-squared test, b) Independent sample t-test, c) Mann-Whitney Test. Significance (*p <* 0.05) is denoted with *.

### Reliability of the Survey

Cronbach’s alpha test results showed that survey items were found to have a good level of reliability. The Cronbach alpha values were as follows: perceived as advantages and disadvantages (0.66), perceived access for patients/customers (0.58), MHPs access (0.66), MHPs satisfaction with telepsychiatry experience (0.89).

### Factors associated with the telepsychiatry practice of MHPs

Correlates of telepsychiatry practice are illustrated in **Table 3**. Telepsychiatry practice was significantly lower among younger MHPs (103; 56.3%) than older ones aged 30-39 years (87; 75.7%) and MHPs aged more than 40 years (45; 63.4%), p = 0.003. The gender and profession of the MHPs were not significantly associated with their telepsychiatry practice. Married MHPs practised a significantly greater amount of telepsychiatry (127; 72.2%) than single MHPs (108; 56%), p = 0.001. The practice of telepsychiatry was highest in the Central Region (88; 73.9%), p = 0.0016. MHPs working in the public sector were shown to be the least in telepsychiatry practice (138; 58%) compared to those working in the private sector (40; 72.7%) or in both sectors (57;75%), p = 0.009. MHPs with shorter duration of work experience (0-2 years) had the lowest in telepsychiatry practice (59; 50%), p = 0.003. The further analysis excluded the 134 MHPs who had no previous experience practising telepsychiatry. Only the 235 MHPs who had previously practised telepsychiatry were included in the following analysis.

**Table 3:** MHPs’ correlates of previous experience of telepsychiatry for treating their patients (n = 369).

Characteristic (N = 369)	Level	Not practicing telepsychiatry n (%) 134 (36.3)	Practicing telepsychiatry. n (%) 235 (63.7)	p value
Age	< 30 years	80 (43.7)	103 (56.3)	*p* = 0.003*
	30-39 years	28 (24.3)	87 (75.7)	
	> 40 years	26 (36.6)	45 (63.4)	
Gender	Male	70 (35.2)	129 (64.8)	*p* = 0.623
	Female	64 (37.6)	106 (62.4)	
Marital status	Single	85 (44.0)	108 (56.0)	*p* = 0.001*
	Married	49 (27.8)	127 (72.2)	
Work region	Central Region	31 (26.1)	88 (73.9)	*p* = 0.016*
	Western Region	36 (48.0)	39 (52.0)	
	Eastern Region	32 (43.8)	41 (56.2)	
	Southern Region	25 (36.2)	44 (63.8)	
	Northern region	10 (30.3)	23 (69.7)	
Profession	Psychiatric consultant	13 (27.1)	35 (72.9)	*p* = 0.254
	Psychiatric specialist	13 (27.1)	35 (72.9)	
	Psychiatric resident	50 (40.0)	75 (60.0)	
	Psychologist	30 (36.6)	52 (63.4)	
	Social worker	28 (42.4)	38 (57.6)	
Physicians	Physician	77 (34.7)	145 (65.3)	*p* = 0.424
	Non-physician	57 (38.8)	90 (61.2)	
Place of work	Public	100 (42.0)	138 (58.0)	*p* = 0.009*
	Private	15 (27.3)	40 (72.7)	
	Both	19 (25.0)	57 (75.0)	
Years of experience in psychiatry	0-2 years	59 (50.0)	59 (50.0)	*p* = 0.003 ^*^
	3-5 years	37 (29.8)	87 (70.2)	
	6-10 years	16 (29.1)	39 (70.9)	
	11+ years	22 (30.6)	50 (69.4)	

Significance (*p <* 0.05) denoted with *.

### Experience of telepsychiatry practice among MHPs

The type of platform used, duration of telepsychiatry practice, and previous self-education of telepsychiatry were not found to be significantly associated with the profession of the MHP. Concerning expertise in telepsychiatry, in general, physicians had treated more patients via telepsychiatry than non-physicians, where 42.8% (62) of physicians had significantly treated more than 21 patients (62; 42.8%), while 37.8% (34) of non-physicians treated fewer than 6 patients through telepsychiatry sessions. During the past year, 41 (28.3%) physicians treated more than 20 patients remotely compared to only 15 (16.7%) non-physicians who had a similar experience. About the number of telepsychiatry sessions per month, physicians conducted a higher number of sessions per month than non-physicians. Surprisingly, non-physicians received significantly more professional training in telepsychiatry than physicians (57; 63.3% vs. 72; 49.7%). Conversely, physicians participated more in telepsychiatry-related research than non-physicians (72; 49.7% vs. 57; 63.3%) **Table 4**.

**Table 4:** Telepsychiatry practice of physician and non-physician MHPs (n =235).

Characteristic (N = 235)	Level	Total n (%)	Physicians n (%) 145 (61.5)	Non-Physicians n (%) 90 (38.3)	p value
Platform used	A (Ayadi)	18 (7.7)	9 (6.3)	9 (10.0)	*p* = 0.179
	L (Labayh)	51 (21.8)	31 (21.5)	20 (22.2)	
	M (Mind)	38 (16.2)	19 (13.2)	19 (21.1)	
	O (Own website)	68 (29.1)	48 (33.3)	20 (22.2)	
	Q (Qarebon)	20 (8.5)	10 (6.9)	10 (11.1)	
	Other	39 (16.7)	27 (18.8)	12 (13.3)	
Duration of telepsychiatry practice	< one year	92 (39.1)	57 (39.3)	35 (38.9)	*p* = 0.867
	1-3 years	103 (43.8)	62 (42.8)	41 (45.6)	
	> three years	40 (17.0)	26 (17.9)	14 (15.5)	
Total number of treated patients	< 6	67 (28.5)	33 (22.8)	34 (37.8)	*p* = 0.001*
	6-10	45 (19.1)	26 (17.9)	19 (21.1)	
	11-16	25 (10.6)	11 (7.6)	14 (15.6)	
	17-21	21 (8.9)	13 (9.0)	8 (8.9)	
	> 21	77 (32.8)	62 (42.8)	15 (16.7)	
Number of patients treated in the past year	< 6	85 (36.2)	42 (29.0)	43 (47.8)	*p* = 0.003*
	6-10	51 (21.7)	29 (20.0)	22 (24.4)	
	11-20	47 (20.0)	33 (22.8)	14 (15.6)	
	> 20	52 (22.1)	41 (28.3)	11 (12.2)	
Number of sessions/months	< 3	88 (37.4)	52 (35.9)	36 (40.0)	*p* = 0.042*
	3-5	66 (28.1)	33 (22.8)	33 (36.7)	
	6-10	40 (17.0)	28 (19.3)	12 (13.3)	
	11-20	23 (9.8)	18 (12.4)	5 (5.6)	
	> 20	18 (7.7)	14 (9.7)	4 (4.4)	
Self-education of telepsychiatry	Yes	189 (80.4)	115 (79.3)	74 (82.2)	*p* = 0.584
	No	46 (19.6)	30 (20.7)	16 (17.8)	
Received professional training in telepsychiatry	Yes	129 (54.9)	72 (49.7)	57 (63.3)	*p* = 0.041*
	No	106 (45.1)	73 (50.3)	33 (36.7)	
Participated in research related to telepsychiatry	Yes	108 (46.0)	59 (40.7)	49 (54.4)	*p* = 0.040*
	No	127 (54.0)	86 (59.3)	41 (45.6)	

Significance (*p <* 0.05) is denoted with *.

### Perceptions, perceived access, and satisfaction of MHPs with telepsychiatry

As for perceived advantages and disadvantages, they mostly agreed that telepsychiatry saved patients’ time and reduced the costs associated with the mental health service. Concerning access to telepsychiatry, perceived patient access was the best-perceived domain, with a mean score of 75.2 ± 17.4. Whereas, concerning providers’ access, this domain showed the least level of agreement (46.6 ± 11.7). However, they mostly agreed that the system used is accessible and easy to use. Satisfaction with the MHPs’ experience of telepsychiatry was moderate (57.6 ± 9.6). They mostly agreed that there is a need for training; they will continue to use it, and they would recommend it to other colleagues **Table 5**.

**Table 5:** Perception, access, and satisfaction of MHPs with telepsychiatry.

Statements (N = 235)	Agree/strongly agree. n (%)	Neutral n (%)	Disagree/strongly disagree. n (%)	Mean (SD)
Perceived advantages and disadvantages for doctors and patients				
All types of patients/customers and diagnoses are suitable for telepsychiatry treatment	103 (43.8)	52 (22.1)	80 (34.1)	2.1 (1.4)
Telepsychiatry is suitable for all stages of treatment	81 (34.5)	58 (24.7)	96 (40.8)	1.8 (1.3)
Using telepsychiatry takes longer than a face-to-face session ¥	103 (43.8)	69 (29.4)	66 (26.8)	2.1 (1.2)
Telepsychiatry session saved my patients/customers’ time	204 (86.8)	23 (9.8)	8 (3.4)	3.2 (0.8)
Workload in local clinics improved with the use of telepsychiatry	183 (77.9)	35 (14.9)	17 (7.2)	2.9 (0.9)
Use of telepsychiatry reduced expenses and costs of service	187 (79.5)	34 (14.5)	14 (6.0)	3.1 (1.0)
Total mean score: Mean (SD)				15 (3.3)
Total mean score percent: Mean (SD)				62.6 (13.9)
Perceived access for patients				
Telepsychiatry sessions allowed my patients or customers to access services earlier than they could have in person	199 (84.6)	22 (9.5)	14 (5.9)	3.1(0.9)
Use of telepsychiatry helped to overcome the cultural and language barriers	162 (69.0)	49 (20.8)	24 (10.2)	2.7 (1.0)
A telepsychiatry session may have made it easier for my patient to get healthcare	205 (87.3)	20 (8.5)	10 (4.2)	3.2 (0.8)
Total mean score: Mean (SD)				9.0 (2.1)
Total mean score percent: Mean (SD)				75.2 (17.4)
Perceived accessibility and access satisfaction				
Technical difficulties made this process too time consuming ¥	136 (57.9)	62 (26.4)	37 (15.7)	2.6 (1.2)
Capable and trained staff were available to provide telepsychiatry services	147 (62.5)	47 (20.0)	41 (17.5)	2.6 (1.2)
I was satisfied with the quality of the picture and audio	170 (72.4)	41 (17.4)	24 (10.2)	2.9 (1.1)
The technology (the normal operation of the instrument rather than any problems encountered) distracted me from the session ¥	123 (52.3)	63 (26.8)	49 (20.9)	2.4 (1.2)
If I had any problems with the telepsychiatry equipment, someone was available to help me	133 (56.5)	50 (21.4)	52 (22.1)	2.4 (1.2)
Overall, the system was accessible and easy to use	195 (83.0)	24 (10.2)	16 (6.9)	3.1 (1.0)
Total mean score: Mean (SD)				11.2 (2.8)
Total mean score percent: Mean (SD)				46.6 (11.7)
Telepsychiatry practice satisfaction				
Telepsychiatry sessions made it easier for me to provide psychiatric services	188 (80.0)	28 (11.9)	19 (8.1)	3.1 (1.0)
The provider-patient rapport was unimpaired using telepsychiatry	150 (63.8)	55 (23.4)	30 (12.8)	2.6 (1.1)
My communication with my patient/customers and/or referring health provider was unimpaired by telepsychiatry	139 (59.2)	63 (26.7)	33 (14.1)	2.6 (1.2)
My patients and customers seemed satisfied with the telepsychiatry sessions	188 (80.0)	34 (14.5)	13 (5.6)	3.0 (0.9)
The inability to touch my patients/customers impaired the diagnosis ¥	124 (52.7)	69 (29.4)	42 (17.9)	2.4 (1.2)
I could accurately assess audible symptoms	173 (73.7)	43 (18.3)	19 (8.1)	2.9 (1.0)
I was unable to observe details of my patient’s facial expression and body movements that would have been important in connecting with him/her ¥	162 (68.9)	58 (24.7)	15 (6.4)	2.9 (1.0)
Telepsychiatry sessions may have improved my patients/customers prognosis	168 (71.5)	47 (20.0)	20 (8.6)	2.8 (1.0)
Telepsychiatry improves clinical efficiency	170 (72.3)	46 (19.6)	19 (8.1)	2.8 (1.0)
I would have preferred to see my patients and customers in person ¥	165 (70.2)	58 (24.7)	12 (5.1)	3.0 (1.0)
There is a need for specific training/expertise in order to practice telepsychiatry ¥	184 (78.3)	32 (13.6)	19 (8.1)	3.1 (1.1)
I did perceive ethical, moral or legal problems associated with practicing telepsychiatry ¥	126(53.7)	63 (26.8)	46 (19.5)	2.5 (1.2)
Overall, I was satisfied with telepsychiatry sessions	190 (80.8)	29 (12.3)	16 (6.9)	3.1 (0.9)
I would use telepsychiatry to see patients and customers again	193 (82.1)	28 (11.9)	14 (6.0)	3.1 (1.0)
I would recommend telepsychiatry to my colleagues	192 (81.7)	31 (13.2)	12 (5.1)	3.2 (1.0)
Total mean score: Mean (SD)				34.6 (5.7)
Total mean score percent: Mean (SD)				57.6 (9.6)

¥ Negative statements, where high scores imply dissatisfaction or perceived problems with telepsychiatry.

## Discussion

Telepsychiatry is a contemporary method of delivering mental health services that is gaining global attention, especially in the post-COVID-19 era (24). The purpose of this study was to assess Saudi MHPs’ awareness and practices of telepsychiatry and their correlates. Additionally, their perceptions and satisfaction with the use of telepsychiatry were also assessed. Telepsychiatry is an increasingly recognized method for delivering mental health services, particularly in the post-COVID-19 era, where its adoption has accelerated globally. Our findings revealed that the majority of MHPs (73.8%) demonstrated awareness of telepsychiatry, with 63.7% of those aware having practical experience in its use. However, the extent of telepsychiatry practice varied significantly across demographic and professional groups, with younger MHPs, those in the Northern Region, and professionals with fewer years of experience being less likely to engage in telepsychiatry. Perceptions of telepsychiatry were generally positive, as MHPs appreciated its ability to save time, reduce costs, and improve patient access to care, with mean percentage scores indicating high agreement in these domains (62.6% for perceived advantages and disadvantages, and 75.2% for perceived patient access). Despite these favorable perceptions, satisfaction with their telepsychiatry practice was moderate (57.6%), with notable concerns about technical difficulties, communication barriers, and ethical issues

### Awareness and practice of telepsychiatry

Our findings showed that most MHPs (73.8%) were aware of telepsychiatry, and nearly half of them (63.7%) had already practiced telepsychiatry with their patients. Awareness and practice rates of telepsychiatry didn’t differ among physicians and non-physicians. In contrast, low levels of knowledge regarding telemedicine technology and telepsychiatry were previously reported in KSA (30, 31) and Poland (32). This may be attributed to limited workshops, seminars, conferences and training that discuss and introduce telemedicine as a vital tool to improve health care services and quality. However, as it is an evolving topic, telehealth knowledge is expected to increase every day.

The awareness of telepsychiatry in our study was higher among older MHPs with longer years of experience. This was in line with the findings of a similar study conducted in KSA (30) that highlighted clinicians’ age and years of experience, which significantly correlated with telepsychiatry awareness and knowledge. This finding could be explained by the fact that longer practice years are usually associated with deeper levels of studying, continuous medical training and checking ongoing research updates in the field of interest. Moreover, our study findings highlighted the existence of a significant difference among different regions of Saudi Arabia regarding the awareness and familiarity with telepsychiatry, as 40% of MHPs from the Northern Region reported being unaware of telepsychiatry. This finding may be indicative of maldistribution of clinical resources and personnel and uneven internet access between regions and between urban/rural areas in KSA (33). Sharing expertise, continuous medical educational programs and data records among different geographical regions would be the ideal approach to overcome the self-limiting national geographic barrier and guarantee full services for different regions of KSA. There were no other significant correlations between awareness of telepsychiatry and sociodemographic factors, including gender, marital status and working sector, similar to the findings reported by a Saudi Arabian study conducted by Khalil et al. (34).

### Perceptions of telepsychiatry

Interestingly, the studied MHPs from KSA mostly showed positive perceptions regarding telepsychiatry practice. They mostly appreciated this method as one for saving time and money, and were least enthusiastic about its suitability for all stages of mental health problems. Similar views and perceptions regarding saving time, effort, and money were revealed in the results of similar studies conducted in KSA (30, 31). Favorable attitudes towards telepsychiatry among MHPs vary across different studies conducted in different countries. It was high among Saudi (31), Spanish (35, 36), and Indian MHPs (37). However, it was lower among Ethiopian MHPs (29). Several factors could stand behind such differences. First, using different assessment tools and the questions that were used to assess attitudes or perceptions. Second, the characteristics and expertise of the sampled MHPs, in addition to their digital literacy. The COVID-19 lockdown greatly impacted providers’ perception and practice of telemedicine (24). Hence, the timing of when the studies were conducted could yield different results depending on whether the study was carried out before, during, or after the COVID-19 era.

In our study, MHPs showed a high perception of telepsychiatry in terms of improving patients’ access to care. Such a high perception regarding improved access to healthcare using telehealth was reported in previous studies conducted in KSA (31) and India (38). The results documented that most health providers expressed that telehealth enhances patients’ direct access to healthcare, especially in cases of emergencies and chronic physical or mental illnesses.

### Satisfaction with telepsychiatry practice

Furthermore, our study revealed that satisfaction with telepsychiatry practice experience was lower in general than the perception of its advantages and improved access for patients. This could be interpreted considering their lower perception regarding their perceived satisfaction with telepsychiatry access and perceived technical difficulties. This is also evident in their highly perceived need for telepsychiatry training and expertise. These findings align with the findings of a similar study that demonstrated a significant association between a favorable attitude towards telepsychiatry and providers’ expertise in using health technology (29).

According to our results, communication with patients, rapport building, preference for in-person consultations, and ethical and moral issues associated with patient management using telepsychiatry were the least satisfactory aspects of their telepsychiatry practice. This is similar to findings of previous research, which showed that professional healthcare providers used to prefer in-person visits, and that patient management typically improves with time and frequent use of telepsychiatry (39).

Eventually, MHPs expressed their willingness to continue using telepsychiatry and to recommend it to their colleagues. This was similar to the findings of Saleh et al. (30), who reported that 89% of MHPs demonstrated willingness to launch telepsychiatry technology at their current workplace, believing that their colleagues would be willing to implement this method (85.7%). Furthermore, they believed telepsychiatry could be integrated into the current medical care system (84.8%). An earlier study by Shittu et al. (40) stated that the willingness of healthcare workers towards telemedicine depends on their current knowledge regarding telehealth applications, the perception of telehealth benefits, and reduced barriers to use.

Though MHP providers agreed that they would continue to use telepsychiatry with patients and would recommend it to colleagues, they also showed high agreement about the need for relevant training in line with the findings reported in a similar study conducted in KSA (30). This finding highlights the emerging need for expanding MHPs’ training activities and providing different telehealth platforms and applications to maximize their benefit in saving time and money and improving access for patients. Such trainings should be expanded to include public hospitals where the practice of telepsychiatry is low and among young junior MHPs who are least likely to use this technology, where they need to be well-prepared for electronic health technologies for future applications. Additionally, more efforts are needed to improve patients’ awareness of the availability of the service. The importance of such training was highlighted in previous studies conducted in Ethiopia (29) and Pakistan (41), predicting that telepsychiatry training would result in significantly improved MHPs’ attitude towards telepsychiatry in addition to equipping the healthcare workplace with the needed technology and personnel.

## Limitations of the study

Our study is a national-level assessment covering all regions of Saudi Arabia, providing a broad perspective on telepsychiatry practices. It includes diverse MHPs, both physicians and non-physicians, contributing valuable insights to the limited body of research on telepsychiatry in the country. However, several limitations must be acknowledged. First, while the survey instrument was developed based on a comprehensive literature review and refined through expert consultations, it was not a fully validated tool, which may have introduced variability in responses. The relatively low Cronbach’s alpha values for some domains, such as perceived patient access (0.58) and MHPs’ access (0.66), suggest moderate internal consistency that could have affected the reliability of the findings. Additionally, approximately 65% of participants worked in the public sector, leading to an underrepresentation of private sector professionals. Psychologists and social workers were also less represented compared to physicians, limiting our ability to capture insights from a broader mental health workforce. This may affect the generalizability of our findings, particularly regarding resource distribution and knowledge accessibility among different MHPs. Furthermore, the study did not explicitly explore key aspects such as specific barriers to telepsychiatry practice, the digital skills of MHPs, the platforms and applications used, or the advantages and disadvantages of each platform. Lastly, as an online survey, participation was inherently limited to MHPs with sufficient digital literacy, introducing potential selection bias. However, recent statistics revealed that nearly 100% of the Saudi population uses the internet, which reduces the risk of selection bias.

## Conclusions and recommendations

The findings of this study highlight the need for strategic action to integrate telepsychiatry more effectively into mental healthcare systems in KSA. While MHPs demonstrated a strong awareness of telepsychiatry and recognized its potential benefits, there remains a gap in consistent adoption and utilization. This underscores the importance of targeted capacity-building initiatives to equip MHPs with the necessary skills and confidence to leverage telepsychiatry tools effectively. Policymakers and stakeholders should prioritize investments in digital infrastructure, including reliable internet access and user-friendly e-health platforms, particularly in underserved regions. Additionally, tailored training programs should be developed to address the specific needs of diverse MHP groups, such as junior professionals and non-physician providers, ensuring equitable access to telepsychiatry resources. To foster widespread acceptance and long-term sustainability, ethical, legal, and regulatory frameworks must be established to guide telepsychiatry practice. By addressing these systemic challenges, KSA can enhance the accessibility and quality of mental healthcare services, ultimately improving outcomes for patients and reducing disparities across regions. Further research is needed to identify barriers to telepsychiatry adoption and assess the impact of policy interventions on practice integration. Future studies should explore user acceptance using theoretical models such as the Unified Theory of Acceptance and Use of Technology (UTAUT) (42) and the Theory of Planned Behavior (TPB) (43).

## Data Availability

The original contributions presented in the study are included in the article/**Supplementary Material**. Further inquiries can be directed to the corresponding author/s.
