# Supplementary material for: Assessment of awareness, practices, perceptions, and satisfaction of telepsychiatry among mental healthcare providers in Saudi Arabia
Source: Front Psychiatry. 2025 May 2;16:1426998. doi: 10.3389/fpsyt.2025.1426998 (PMC12081428; doi:10.3389/fpsyt.2025.1426998)
Supplement: Supplementary file 1 [file DataSheet1.pdf]

Assessment of awareness, practices, perceptions, and satisfaction of telepsychiatry among mental healthcare providers in Saudi Arabia

Abstract

Informed Consent

1. Do you agree to participate in this study? *(Mark only one oval)*
    - Yes
    - No

Email (Optional): ___________________________

Data Collectors

2. Data collector number: *(Mark only one oval)*

Section 1: Personal Information

3. Gender: *(Mark only one oval)*
    - Male
    - Female
4. Age: *(Please write it in numbers)*
   Answer: ___________________________
5. Marital Status: *(Mark only one oval)*
    - Single
    - Married
6. Work Region: *(Mark only one oval)*
    - Eastern Region
    - Southern Region
    - Northern Region
    - Western Region
    - Central Region
7. Place of Work: *(Mark only one oval)*
    - Public

- Private
- Both

8. Position: *(Mark only one oval)*
    - Psychiatric Resident
    - Psychiatric Specialist
    - Psychiatric Consultant
    - Psychologist
    - Social Worker
    - Other: ___________________________

9. Years of Experience in the Mental Health Field: *(Please write it in numbers)*
   Answer: ___________________________

Section 2: Awareness of Tele-psychiatry

10. Have you heard or had any prior knowledge regarding Tele-psychiatry? *(Mark only one oval)*
    - Yes
    - No

Section 3: Awareness of Telepsychiatry (Part 1)

11. Do you have any experience in treatment (patients/customers) via Tele-psychiatry (video, telephone, chat, text, etc.)? *(Mark only one oval)*
    - Yes
    - No

(If "Yes" above)
12. Which platform(s) have you used? *(Mark all that apply)*
- [ ] Mind
- [ ] Labayh
- [ ] Qarebon
- [ ] Ayadi
- [ ] My own Website
- [ ] Other: ___________________________

13. How many patients/customers have you treated through Tele-psychiatry? *(Mark only one oval)*

- Less than 6
- 6–10
- 11–16
- 17–21
- More than 21

14. For how many years have you been using Tele-psychiatry? *(Mark only one oval)*
    - Less than one year
    - 1–3 years
    - More than 3 years
15. How many patients/customers over the past year? *(Mark only one oval)*
    - Less than 6
    - 6–10
    - 10–20
    - More than 20
16. How many sessions per month? *(Mark only one oval)*
    - Less than 3
    - 3–5
    - 6–10
    - 11–20
    - More than 20
17. Have you read any professional resource on this method? *(Mark only one oval)*
    - Yes
    - No

Section 4: Access and Satisfaction of Tele-psychiatry

18. Have you undergone any professional training regarding Tele-psychiatry? *(Mark only one oval)*
    - Yes

- No

19. Have you ever joined any research related to Tele-psychiatry? *(Mark only one oval)*
    - Yes
    - No
20. Please indicate your level of agreement with the following statements: *(Check one box for each statement)*

Statement	Strongly Agree	Agree	Neither Agree nor Disagree	Disagree	Strongly Disagree
All types of patients/customers and diagnoses are suitable for Tele-psychiatry treatment.	[ ]	[ ]	[ ]	[ ]	[ ]
Tele-psychiatry is suitable for all stages of treatment.	[ ]	[ ]	[ ]	[ ]	[ ]
Using Tele-psychiatry takes longer than a face-to-face session.	[ ]	[ ]	[ ]	[ ]	[ ]
Tele-psychiatry sessions saved my patients/customers time.	[ ]	[ ]	[ ]	[ ]	[ ]
Tele-psychiatry sessions allowed my patients/customers to access services earlier than in person.	[ ]	[ ]	[ ]	[ ]	[ ]
Technical difficulties made this process too time-consuming.	[ ]	[ ]	[ ]	[ ]	[ ]
Workload in local clinics improved by the use of Tele-psychiatry.	[ ]	[ ]	[ ]	[ ]	[ ]

Statement	Strongly Agree	Agree	Neither Agree nor Disagree	Disagree	Strongly Disagree
Use of Tele-psychiatry helped to overcome cultural and language barriers.	[ ]	[ ]	[ ]	[ ]	[ ]
Tele-psychiatry sessions may have made it easier for my patient to get healthcare.	[ ]	[ ]	[ ]	[ ]	[ ]
Tele-psychiatry sessions made it easier for me to provide psychiatric services.	[ ]	[ ]	[ ]	[ ]	[ ]
Capable and trained staff was available to provide Tele-psychiatry services.	[ ]	[ ]	[ ]	[ ]	[ ]
Use of Tele-psychiatry reduced expenses and costs of services.	[ ]	[ ]	[ ]	[ ]	[ ]
I was satisfied with the quality of the picture and audio.	[ ]	[ ]	[ ]	[ ]	[ ]
The technology distracted me from the session.	[ ]	[ ]	[ ]	[ ]	[ ]
If I had any problems with the Tele-psychiatry equipment, someone was available to help me.	[ ]	[ ]	[ ]	[ ]	[ ]
Overall, the system was accessible and easy to use.	[ ]	[ ]	[ ]	[ ]	[ ]
The provider-patient rapport was unimpaired using Tele-psychiatry.	[ ]	[ ]	[ ]	[ ]	[ ]

Statement	Strongly Agree	Agree	Neither Agree nor Disagree	Disagree	Strongly Disagree
My communication with my patient/customers and/or referring health provider was unimpaired.	[ ]	[ ]	[ ]	[ ]	[ ]
My patients/customers seemed satisfied with Tele-psychiatry sessions.	[ ]	[ ]	[ ]	[ ]	[ ]
The inability to touch my patients/customers impaired the diagnosis.	[ ]	[ ]	[ ]	[ ]	[ ]
I could accurately assess audible symptoms.	[ ]	[ ]	[ ]	[ ]	[ ]
I was unable to observe details of my patient's facial expression and body movements.	[ ]	[ ]	[ ]	[ ]	[ ]
Tele-psychiatry sessions may have improved my patients/customers prognosis.	[ ]	[ ]	[ ]	[ ]	[ ]
Tele-psychiatry improves clinical efficiency.	[ ]	[ ]	[ ]	[ ]	[ ]
I would have preferred to see my patients/customers in person.	[ ]	[ ]	[ ]	[ ]	[ ]
There is a need for specific training/expertise to practice Tele-psychiatry.	[ ]	[ ]	[ ]	[ ]	[ ]

Statement	Strongly Agree	Agree	Neither Agree nor Disagree	Disagree	Strongly Disagree
I perceived ethical/moral/legal problems associated with practicing Tele-psychiatry.	[ ]	[ ]	[ ]	[ ]	[ ]
Overall, I was satisfied with the Tele-psychiatry session.	[ ]	[ ]	[ ]	[ ]	[ ]
I would use Tele-psychiatry to see patients/customers again.	[ ]	[ ]	[ ]	[ ]	[ ]
I would recommend Tele-psychiatry to my colleagues.	[ ]	[ ]	[ ]	[ ]	[ ]
